# A novel allograft combined with modified purse-string suture fixation for annulus fibrosus repair: biomechanical evaluation in a porcine lumbar disc model

**DOI:** 10.3389/fbioe.2026.1738755

**Published:** 2026-09-28

**Authors:** Yueh-Ying Hsieh, Lien-Chen Wu, Fon-Yih Tsuang, Che-Yung Kuan, Chang-Jung Chiang

**Affiliations:** 1 Department of Orthopedics, Shuang Ho Hospital, Taipei Medical University, New Taipei, Taiwan; 2 Department of Orthopedics, Hsin Kuo Min Hospital, Taipei Medical University, Taoyuan, Taiwan; 3 Department of Orthopedics, School of Medicine, College of Medicine, Taipei Medical University, Taipei, Taiwan; 4 Department of Orthopedic Surgery, Wan Fang Hospital, Taipei Medical University, Taipei, Taiwan; 5 Graduate Institute of Biomedical Materials and Tissue Engineering, College of Biomedical Engineering, Taipei Medical University, Taipei, Taiwan; 6 Division of Neurosurgery, Department of Surgery, National Taiwan University Hospital, Taipei, Taiwan

**Keywords:** annular repair, biomechanical evaluation, herniated discs, MPSS technique, truncated-conical allograft

## Abstract

**Background:**

Annulus fibrosus (AF) defects may permit recurrent nucleus pulposus extrusion and are difficult to repair because of limited vascularity and persistent mechanical loading. Commercial bone-anchored annular closure devices are intended to occlude large post-discectomy defects, whereas tissue-derived grafts may provide an alternative defect-filling strategy. However, the immediate sealing performance of allograft-based AF repair remains incompletely characterized.

**Purpose:**

This study developed an allograft designed to stably repair AF defects and evaluated its sealing performance in porcine lumbar discs.

**Methods:**

Thirty porcine lumbar motion segments were allocated to five experimental groups. Group A received a 26-gauge annular puncture injury without repair and served as a puncture-reference condition rather than a defect-matched control. In Groups B1, B2, C1, and C2, a standardized 3-mm annular defect was created to accommodate a 3-mm-diameter allograft. Group B1 received a standard cylindrical allograft, whereas Group B2 received an allograft that expanded asymmetrically after hydration to form a truncated-conical geometry. Groups C1 and C2 received the corresponding allografts secured using modified purse-string suture (MPSS) fixation. Acute sealing performance was evaluated by static leakage testing, and primary statistical comparisons were restricted to Groups B1–C2.

**Results:**

Group A, included descriptively as a puncture-reference condition, had a mean failure pressure of 0.395 ± 0.083 MPa and was excluded from the primary inferential analysis. Among groups with standardized 3-mm defects, mean failure pressures were 0.802 ± 0.334 MPa for the standard cylindrical allograft (Group B1), 1.539 ± 0.334 MPa for the hydration-expanded truncated-conical allograft (Group B2), 2.638 ± 0.386 MPa for the standard cylindrical allograft with MPSS fixation (Group C1), and 2.892 ± 0.685 MPa for the hydration-expanded truncated-conical allograft with MPSS fixation (Group C2). Failure pressure differed significantly among Groups B1–C2 (p < 0.001). MPSS fixation significantly increased failure pressure for both graft configurations, whereas the difference between Groups B1 and B2 did not reach statistical significance after adjustment for multiple comparisons (adjusted p = 0.052).

**Conclusion:**

Among porcine lumbar motion segments with standardized 3-mm AF defects, MPSS fixation significantly increased acute leakage resistance for both graft configurations. The hydration-expanded truncated-conical allograft showed a higher mean failure pressure than the standard cylindrical allograft when used without fixation, although this difference did not reach statistical significance after adjustment for multiple comparisons. These findings support the acute mechanical feasibility of the allograft-MPSS construct.

## 1 Introduction

Intervertebral discs are composed of a central nucleus pulposus (NP) surrounded by the annulus fibrosus (AF). This structure offers flexibility to the spine while also providing support and cushioning during loading. If the AF is perforated, which typically happens through injury or excessive loading, the NP may extrude through the defect and put pressure on the adjacent spinal nerve roots. Herniated discs can cause chronic pain due to nerve stimulation and are difficult to heal because of the limited vascularization and constant mechanical stress.

Autologous grafts generally have low immunogenicity because the tissue is obtained from the same individual. However, harvesting an AF autograft from another intervertebral disc would create unacceptable donor-site damage. Allogeneic AF tissue may therefore provide a practical alternative for defect filling. Nevertheless, the biocompatibility, host response, and long-term integration of an allograft depend on the tissue source and processing method and must be established for the intended application.

Limited discectomy is commonly performed to remove herniated nucleus pulposus tissue while preserving as much native disc tissue as possible (Reheman et al., 2024). However, removal of AF and NP tissue may weaken the motion segment, reduce intervertebral disc height, alter segmental biomechanics, and contribute to recurrent disc herniation (Kursumovic et al., 2019; Zheng et al., 2022).

Recurrent disc herniation has been reported in up to approximately 26% of patients after lumbar discectomy (Musa et al., 2023). Annular closure used as an adjunct to limited discectomy has therefore been investigated as a strategy to reduce the risk of reherniation, particularly in patients with large residual annular defects (Nunley et al., 2023).

A commercially available approach is the Barricaid annular closure device, a permanent bone-anchored implant intended to reduce reherniation and reoperation after primary lumbar discectomy in patients with large annular defects. The device consists of a flexible polymer component that occludes the annular defect and a titanium anchor that secures the construct to the adjacent vertebral body. Its indicated defect dimensions are 4–6 mm in height and 6–10 mm in width. Barricaid therefore primarily functions as a mechanically anchored barrier following discectomy (Ardeshiri et al., 2019; Burkhardt and Oertel, 2021; Kienzler et al., 2021; Nunley et al., 2023; Parker et al., 2016). However, the immediate sealing performance of tissue-derived, non–bone-anchored repair constructs for smaller AF defects remains insufficiently characterized. Such constructs may represent a distinct repair strategy in which the defect is filled with native tissue-derived material and stabilized locally without vertebral fixation.

The 3-mm defect size was selected as a standardized small annular defect that matched the initial diameter of the dehydrated allograft and enabled defect-matched comparisons among the repaired groups. This dimension also has precedent in experimental AF closure models; Bron et al. (2010) used a standardized 3-mm circular AF defect in a goat nucleus-replacement model, and our previous porcine study evaluated graft-based repair of an annular punch defect measuring approximately 3 mm × 4 mm × 11 mm (Yang et al., 2016). Accordingly, the present 3-mm model was intended as a controlled small-defect platform for evaluating a tissue-based, non–bone-anchored repair strategy rather than as a representation of the larger and more irregular defects typically encountered after lumbar discectomy.

The approach investigated in the present study therefore used a tissue-derived allograft to fill a standardized 3-mm AF defect, with or without supplementary modified purse-string suture (MPSS) fixation. No nucleus pulposus removal or vertebral bone anchoring was performed. Accordingly, the aim of this study was to compare the acute sealing performance of different allograft geometries and fixation configurations and to determine whether allograft geometry and MPSS fixation improve resistance to pressure-driven leakage in a porcine lumbar motion-segment model.

## 2 Methods

### 2.1 Specimen preparation

Lumbar spines from six-month-old pigs (80–100 kg) were obtained from a licensed slaughterhouse within 12 h post-mortem. A total of 30 motion segments, each comprising one intervertebral disc and two adjacent intact vertebrae, were isolated. All soft tissues were removed except for anterior and posterior longitudinal ligaments. Specimens were stored in 0.9% saline at 4 °C to maintain hydration.

### 2.2 Specimen standardization

To reduce interspecimen variability, only lumbar motion segments obtained from six-month-old pigs weighing 80–100 kg within 12 h post-mortem were included. All specimens were stored immersed in 0.9% saline at 4 °C before preparation and testing, and all annular injuries or defects were created at the same right anterolateral location. This standardized storage protocol was used to minimize variability related to post-mortem interval, tissue dehydration, injury location, and defect orientation. Specimen-specific tissue water content and AF thickness at the injury site were not measured. Residual anatomical and tissue-property variation was addressed through computer-generated random allocation before creation of the annular injury or defect.

### 2.3 Preparation of allogeneic AF grafts

Fresh AF tissue was harvested as wedge-shaped blocks with a wider outer rim and a narrower inner rim (Figure 1a). After excision, contraction of the denser outer AF reduced the apparent wedge shape. The harvested tissue was decellularized using the previously optimized porcine AF protocol described by Wu et al. (2017). Briefly, the samples were frozen in liquid nitrogen (−196 °C) for 22 h and subsequently thawed at 37 °C for 2 h. The samples were then incubated with agitation in hypotonic buffer containing 10 mM Tris–HCl (pH 8.0), 0.1% EDTA, and 10 KIU/mL aprotinin at 37 °C for 24 h. This was followed by treatment in Tris–HCl buffer containing 0.1% sodium dodecyl sulfate (SDS), 0.1% EDTA, and 10 KIU/mL aprotinin at 4 °C for 24 h. After washing, the tissues were incubated with DNase (50 U/mL) and RNase (1 U/mL) in Tris buffer containing 50 mM Tris–HCl, 10 mM magnesium chloride, and 50 mg/mL bovine serum albumin (pH 7.5) at 37 °C for 3 h with gentle agitation. The tissues were then washed three times in PBS at 37 °C for a total of 8 h.

**FIGURE 1:**
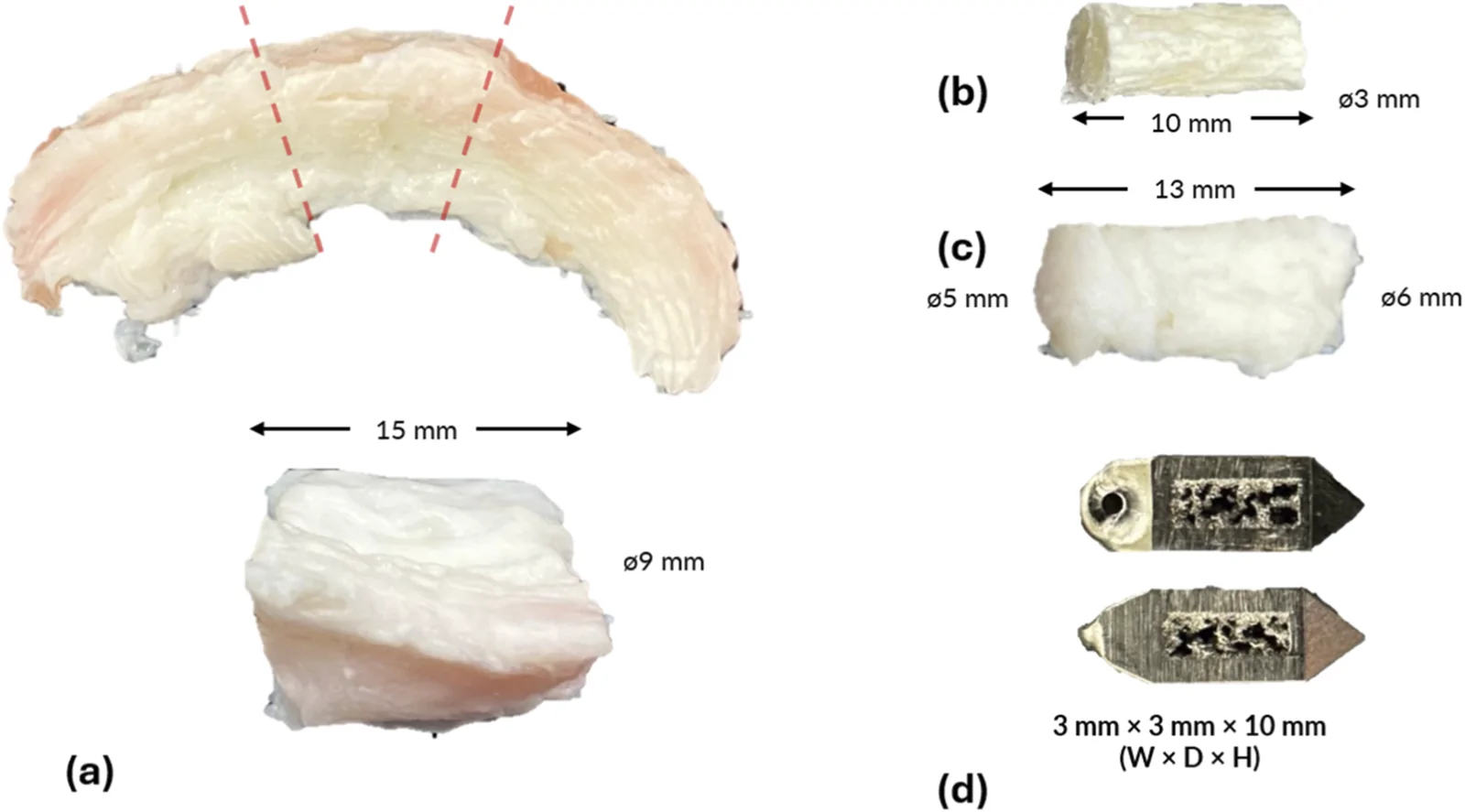
Preparation and configuration of the annulus fibrosus (AF) allograft. **(a)** Fresh AF tissue was harvested as a wedge-shaped block with a wider outer rim and a narrower inner rim. After excision, contraction of the dense outer layer reduced the wedge appearance, whereas the inner portion showed minimal shrinkage. **(b)** The AF tissue was decellularized, dehydrated, and freeze-dried to form an approximately cylindrical plug. **(c)** After hydration, the geometry-modified graft expanded asymmetrically to form a truncated-conical profile with an enlarged nucleus pulposus-facing end. **(d)** The allograft was secured using modified purse-string suture anchors with crossover compression to achieve a near-watertight closure.

Following decellularization, the tissues were dehydrated and freeze-dried to produce approximately cylindrical plugs measuring 3 mm in diameter and 10 mm in length (Figure 1b) (Lin et al., 2016). For the geometry-modified graft, rehydration produced asymmetric expansion: the NP-facing end increased from approximately 3–6 mm in diameter, whereas the outer AF-facing end remained approximately 3 mm in diameter. This expansion resulted in a truncated-conical geometry intended to improve mechanical engagement within the defect. The graft was positioned in a standardized orientation, with the end intended to form the wider hydrated portion directed toward the NP.

The AF graft was secured using the previously reported modified purse-string suture (MPSS) technique without modification to the suturing configuration (Yang et al., 2016). The MPSS construct consisted of two circumferential loop structures with a crossover compression suture across the defect. Four 3D-printed titanium suture anchors were positioned circumferentially around the defect margin, using the same anchor design and material reported previously (Wu et al., 2024). The anchors used in the present study measured 3 mm × 3 mm × 10 mm (Figure 1d), and a USP 3–0 Ethibond® suture (Ethicon, Somerville, NJ, United States) was used throughout all MPSS repairs (Chiang et al., 2012). The only difference from the anchor hardware reported by Wu et al. (2024) was a minor dimensional increase from 2.5 mm × 2.5 mm × 10 mm to 3 mm × 3 mm × 10 mm. No changes were made in the number of anchors, suture arrangement, or crossover compression pattern among the MPSS-treated specimens. Tightening of the construct produced circumferential contraction and crossover compression of the graft within the defect, achieving a near-watertight closure.

### 2.4 Experimental groups and allocation

Motion segments were randomly assigned to five groups (n = 6 per group). After specimen preparation and eligibility assessment, the 30 numbered motion segments were allocated in a 1:1:1:1:1 ratio to Groups A, B1, B2, C1, and C2 (n = 6 per group) using a computer-generated random sequence. Allocation was completed before creation of the annular defect and remained unchanged thereafter.

Group A—Puncture Injury Only: A 26-gauge annular puncture injury was created without repair.

Group B1—Standard Cylindrical Allograft: A standardized 3-mm AF defect was created and filled with a dehydrated standard cylindrical allograft measuring approximately 3 mm in diameter and 10 mm in length (Figure 2a). No anchors or sutures were used.

**FIGURE 2:**
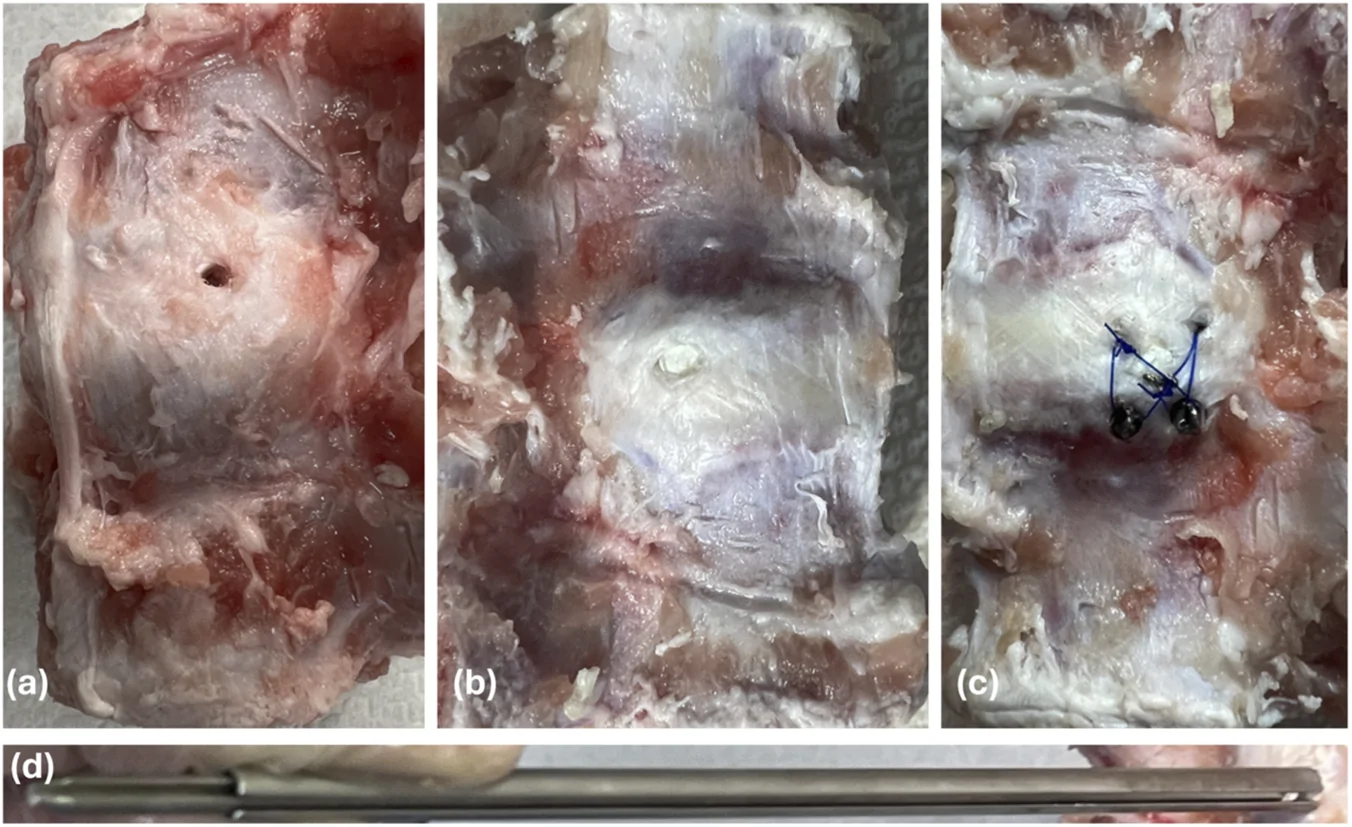
Experimental annulus fibrosus repair groups. Group A received a 26-gauge annular puncture injury without repair. For Groups B1, B2, C1, and C2, **(a)** a standardized 3-mm annular defect was created to accommodate a 3-mm-diameter allograft. **(b)** The defect was filled with either a standard cylindrical allograft (Group B1) or a hydration-expanded truncated-conical allograft (Group B2) without suture fixation. **(c)** The corresponding allografts were secured using the modified purse-string suture technique with circumferential anchors and crossover compression (Groups C1 and C2, respectively). **(d)** A custom-designed cannulated annular defect-preparation guide was used to control defect diameter, depth, orientation, and graft insertion.

Group B2—Hydration-Expanded Truncated-Conical Allograft: A standardized 3-mm AF defect was created and filled with an initially cylindrical dehydrated allograft measuring approximately 3 mm in diameter and 10 mm in length (Figure 2b). After hydration, the NP-facing end expanded to approximately 6 mm, whereas the outer AF-facing end remained approximately 3 mm, resulting in a truncated-conical geometry (Figure 1c). No anchors or sutures were used.

Group C1—Standard Cylindrical Allograft + MPSS: The same standardized 3-mm AF defect and standard cylindrical allograft used in Group B1 were employed, followed by MPSS fixation using four circumferential anchors and crossover compression (Wu et al., 2024).

Group C2—Hydration-Expanded Truncated-Conical Allograft + MPSS: The same standardized 3-mm AF defect and hydration-expanded truncated-conical allograft used in Group B2 were employed, followed by the MPSS fixation procedure used in Group C1, as shown in Figures 2c,d.

### 2.5 Creation of annular injuries and defects

All injuries and defects were created at the right anterolateral aspect of the AF. Group A received a 26-gauge needle puncture injury. The needle was advanced to a prespecified insertion depth of 11–12 mm to penetrate the nucleus pulposus and was then withdrawn. No repair was performed. The resulting injury is reported according to the nominal needle gauge because the residual puncture-opening diameter after elastic recoil of the AF was not independently measured.

For Groups B1, B2, C1, and C2, a standardized circular AF defect with a nominal diameter of 3 mm and depth of 10 mm was created to match the initial 3-mm diameter of the dehydrated allograft and provide a common defect geometry across the repaired groups. A custom-designed cannulated annular defect-preparation guide with an inner diameter of 3 mm, outer diameter of 5 mm, and a longitudinal C-shaped slot was used throughout the procedure. The guide directed a 3-mm drill and limited drill advancement to a nominal depth of 10 mm, thereby standardizing defect location, orientation, diameter, and depth. The same cannulated channel subsequently guided the allograft punch/inserter and maintained alignment during graft placement. The achieved defect dimensions were not independently remeasured after drilling. No discectomy, intentional nucleus pulposus removal, or vertebral bone preparation was performed.

### 2.6 Static leakage test

Static leakage testing was conducted according to the protocol described by Yang et al. (2016). A 0.9 mm stainless steel tube was inserted vertically through the upper vertebra and endplate and into the nucleus pulposus (Figure 3a) and connected to a servo-hydraulic testing machine (MTS 858 Mini Bionix II). Blue-dyed alginate gel was infused at a constant piston displacement rate of 5 mm/min (Figure 3b). The intradiscal pressure (MPa) was calculated by dividing the axial force (N) applied to the piston by the cross-sectional area of the piston (mm^2^). Failure was defined as the first visible extrusion of blue-dyed alginate gel through the annular defect or along the graft–annulus repair interface during direct visual observation. The calculated pressure at that instant was recorded as the failure pressure, and the test was stopped. Thus, the endpoint represented the onset of leakage rather than complete graft extrusion or catastrophic disruption of the motion segment.

**FIGURE 3:**
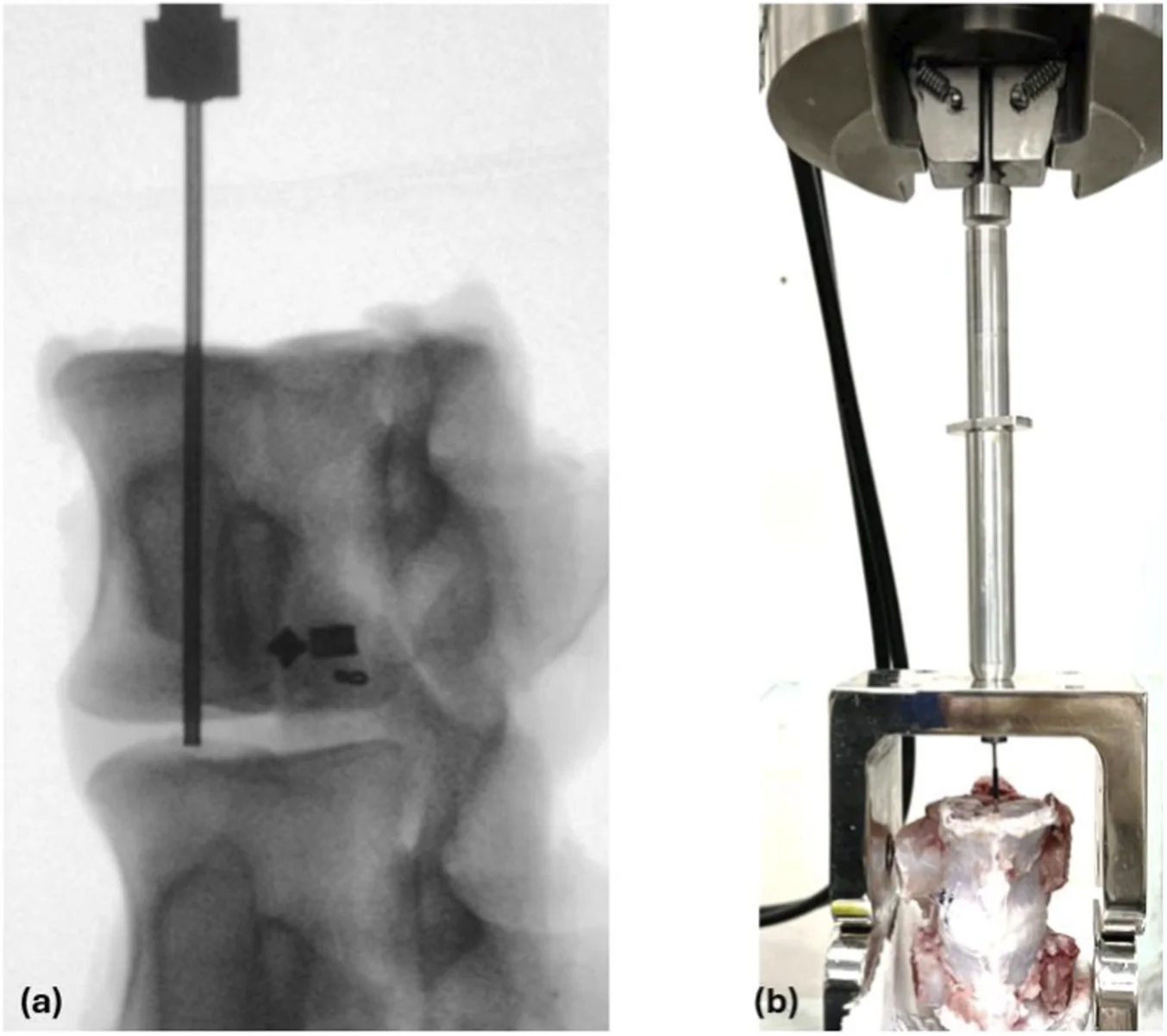
Static leakage-test setup. **(a)** A 0.9-mm stainless steel tube was inserted vertically through the upper vertebral body and endplate into the nucleus pulposus. **(b)** The tube was connected to a stainless steel syringe and piston mounted on a servo-hydraulic testing machine.

Failure-pressure data are presented as the mean ± standard deviation (SD). Because Group A received a 26-gauge puncture injury whereas Groups B1, B2, C1, and C2 received standardized 3-mm AF defects, Group A was treated as a puncture-reference condition and was excluded from the primary inferential analysis. Primary statistical comparisons were therefore restricted to Groups B1, B2, C1, and C2, which shared the same defect-preparation protocol. Differences among the four experimental groups were evaluated using one-way analysis of variance (ANOVA). Following a significant omnibus ANOVA result, all pairwise comparisons among Groups B1–C2 were performed using Tukey’s honestly significant difference (HSD) test to control the family-wise type I error rate. Group A is reported descriptively only. All tests were two-sided, and an adjusted p-value <0.05 was considered statistically significant. Statistical analyses were performed using IBM SPSS Statistics, version 28.0.

## 3 Results

Failure pressure, defined as the pressure at the onset of visible gel leakage through the annular defect or graft–annulus interface (Figure 4), is summarized in Figure 5 and Table 1. Failure pressure differed significantly among the five experimental groups. Group A, which received an unrepaired 26-gauge puncture injury, had a mean failure pressure of 0.395 ± 0.083 MPa. Among specimens with standardized 3-mm AF defects, mean failure pressure was 0.802 ± 0.334 MPa for the standard cylindrical allograft without fixation (Group B1), 1.539 ± 0.334 MPa for the hydration-expanded truncated-conical allograft without fixation (Group B2), 2.638 ± 0.386 MPa for the standard cylindrical allograft with MPSS fixation (Group C1), and 2.892 ± 0.685 MPa for the hydration-expanded truncated-conical allograft with MPSS fixation (Group C2).

**FIGURE 4:**
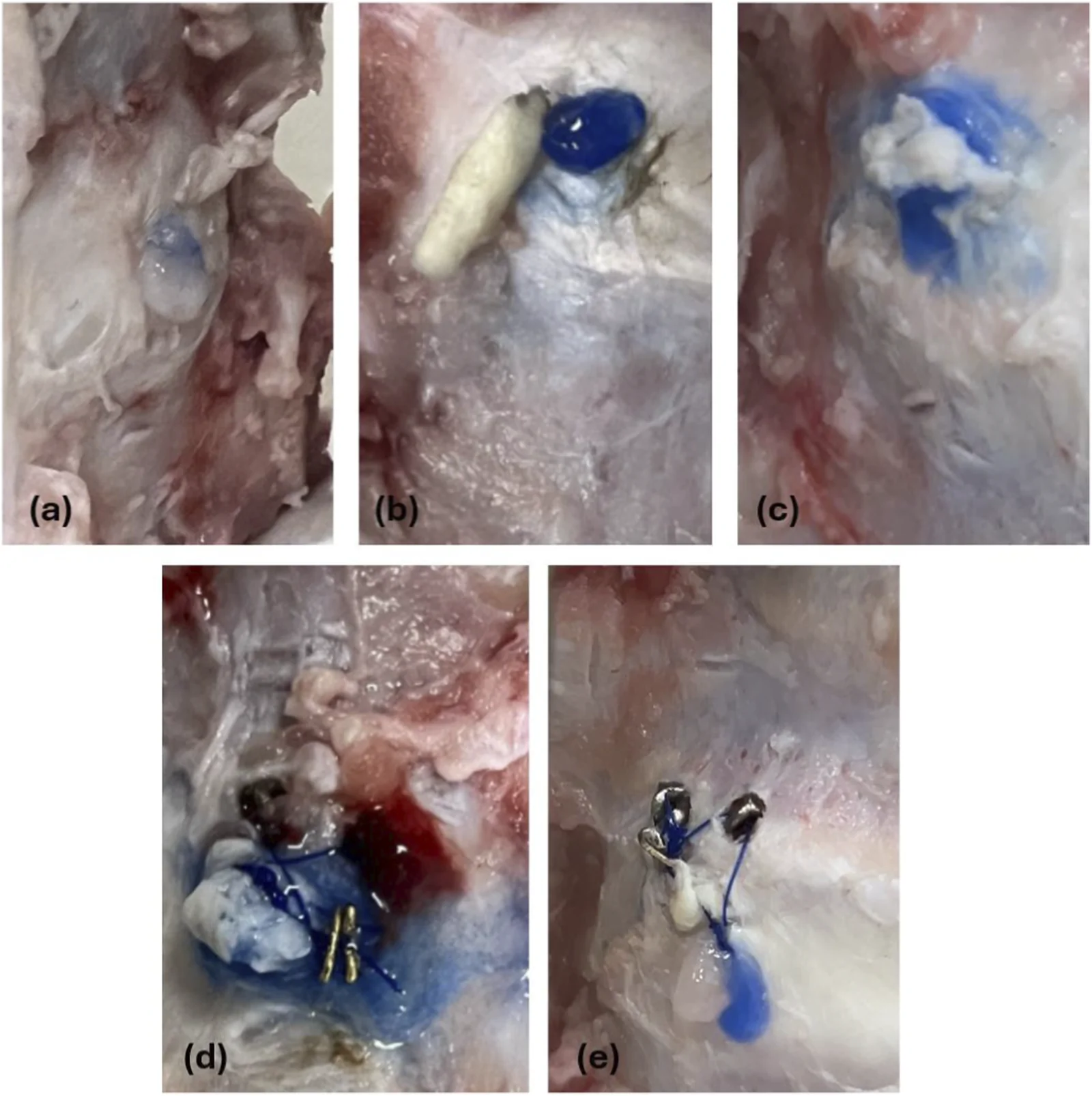
Failure modes observed in each group after leakage testing. **(a)** Annular burst-out in Group A; **(b)** complete graft extrusion in Group B1; **(c)** blue-colored alginate gel leakage in Group B2; **(d)** partial graft extrusion in Group C1; **(e)** graft secured by suture despite blue-colored alginate gel leakage in Group C2.

**FIGURE 5:**
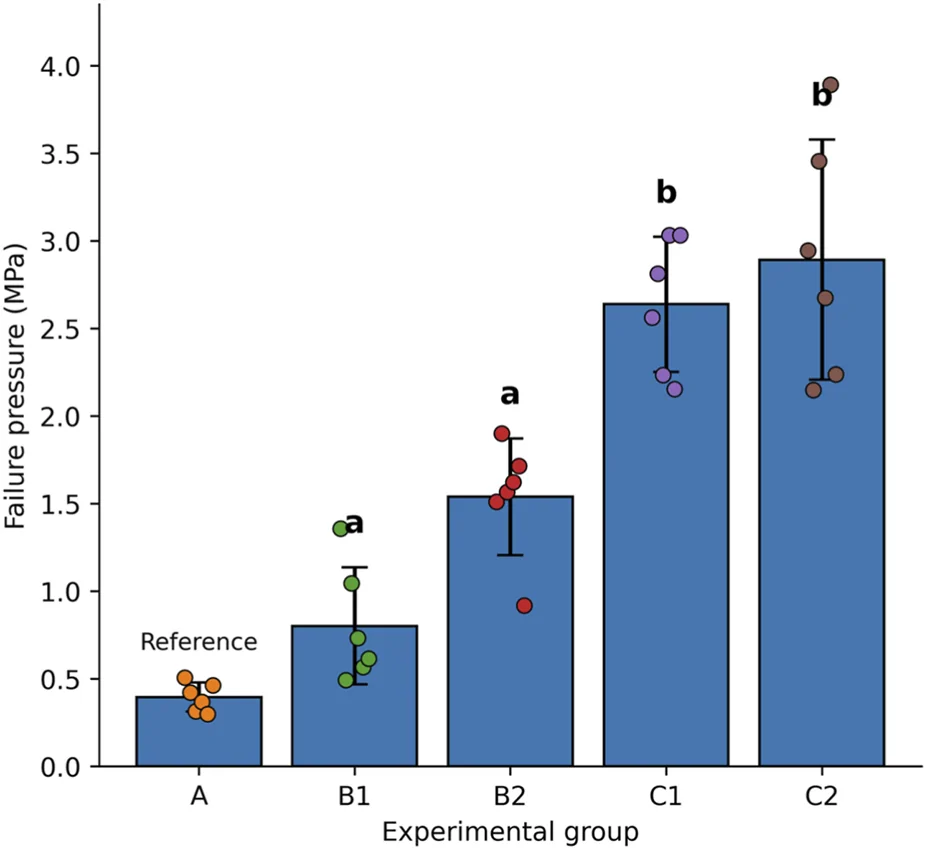
Failure pressure by experimental group. Bars represent mean failure pressure, error bars represent standard deviation, and points represent individual specimens (n = 6 per group). Group A is shown descriptively as a puncture-reference condition and was excluded from the primary inferential analysis because its injury geometry differed from that of Groups B1–C2. For Groups B1–C2, groups without a shared lowercase letter were significantly different according to Tukey’s HSD pairwise comparisons at an adjusted p-value <0.05.

**TABLE 1:** Failure pressure at the onset of gel leakage for individual specimens in each experimental group.

Group	Sample 1	Sample 2	Sample 3	Sample 4	Sample 5	Sample 6	Mean	SD
A	0.506	0.422	0.315	0.367	0.299	0.463	0.395	0.083
B1	1.358	0.492	1.045	0.734	0.567	0.615	0.802	0.334
B2	1.511	1.901	1.566	1.623	1.716	0.918	1.539	0.334
C1	2.562	2.813	2.234	3.033	2.154	3.032	2.638	0.386
C2	2.945	2.149	3.456	2.675	3.892	2.237	2.892	0.685

1) Values are failure pressures at the onset of visible gel leakage, expressed in MPa. SD, standard deviation. 2) Group A represents a puncture-reference condition and was excluded from the primary inferential statistical analysis because its injury geometry differed from that of Groups B1–C2.

Because Group A received a 26-gauge puncture injury rather than the standardized 3-mm AF defect used in Groups B1–C2, Group A was treated as a descriptive puncture-reference condition and was excluded from the primary inferential analysis. Among Groups B1, B2, C1, and C2, one-way ANOVA demonstrated a significant overall difference in failure pressure, F (3, 20) = 27.05, p < 0.001. Tukey’s HSD pairwise comparisons showed that the difference between Groups B1 and B2 did not reach statistical significance (adjusted p = 0.052). Group C1 had a significantly higher failure pressure than Group B1 (adjusted p < 0.001) and Group B2 (adjusted p = 0.003), and Group C2 had a significantly higher failure pressure than Group B1 and Group B2 (both adjusted p < 0.001). No significant difference was observed between Groups C1 and C2 (adjusted p = 0.773). Thus, under the common 3-mm defect configuration, MPSS fixation significantly increased failure pressure for both graft geometries, whereas a statistically significant effect of graft geometry alone was not demonstrated.

## 4 Discussion

When repairing the AF it is important to preserve disc material, which can reduce the risk of reherniation and create a favorable environment for NP replacement. Traditional discectomy not only removes the ruptured portion of the AF, but also removes a portion of the NP to decrease the risk of expulsion during physical loading. If the AF defect could be tightly sealed or even healed after disc herniation, it may be possible to minimize the amount of NP tissue that needed to be removed during the discectomy procedure. Preserving native disc tissue is an important objective of limited discectomy because removal of NP and AF tissue may alter disc height and segmental biomechanics. In the present study, MPSS fixation increased the failure pressure of the hydration-expanded truncated-conical allograft relative to the same graft used without fixation. Group C2 exhibited the highest mean failure pressure among the experimental groups. These findings suggest that the enlarged NP-facing end of the truncated-conical graft acted as an internal mechanical barrier against pressure-driven extrusion, whereas MPSS fixation further stabilized and compressed the graft within the defect. However, because Group A and Groups B1–C2 involved different injury geometries, direct percentage comparisons between the injury-only group and the repaired 3-mm-defect groups should not be interpreted as isolated treatment effects.

Generally, severe disc herniations do not heal naturally without intervention, with most patients requiring surgery. Since the early 2000s, researchers have been assessing different biocompatible materials to effectively seal AF defects, such as non-absorbable polymer-based biomaterials and absorbable collagen–glycosaminoglycan scaffolds (Lee et al., 2021; Long et al., 2016). However, most efforts resulted in material loss, migration, and poor sealing capacity. Previous AF repair strategies have used nonabsorbable polymers, collagen-based scaffolds, sutures, and annular closure devices; however, insufficient sealing and material migration remain important challenges. In the present study, the hydration-expanded truncated-conical graft showed a higher mean failure pressure than the standard cylindrical graft when both were used without suture fixation; however, this difference did not reach statistical significance after restriction of the primary analysis to the defect-matched Groups B1–C2. Thus, the present data suggest a possible mechanical contribution of the enlarged NP-facing end but do not establish a statistically significant independent effect of graft geometry. This finding supports the mechanical contribution of the enlarged NP-facing end, which may function as an internal buttress against pressure-driven graft extrusion. Nevertheless, the present results represent acute static sealing performance and do not establish long-term graft retention, fatigue resistance, biological healing, or host–graft integration.

Suturing the anchors in place significantly increased the maximum pressure able to be borne by the grafts. The MPSS system tightly contracts the allograft into the defect and keeps the suture direction almost parallel to the native collagen-fiber orientation of the AF to minimize tension on the defect edges (Chiang et al., 2012; Yang et al., 2016). MPSS fixation further increased failure pressure for both graft geometries (Wu et al., 2024). Relative to the corresponding unfixed graft groups, mean failure pressure was approximately 229% higher for the standard cylindrical allograft (Group C1 vs. Group B1) and approximately 88% higher for the hydration-expanded truncated-conical allograft (Group C2 vs. Group B2). The MPSS construct compresses the graft within the defect and maintains the suture direction approximately parallel to the native AF collagen-fiber orientation, which may reduce stress concentration at the defect margins. In addition, the 3D-printed anchors used in this study demonstrated substantial pullout resistance in our previous investigation. Bron et al. (2010) reported intradiscal pressures approaching 3 MPa under axial loading in a goat disc model. However, such load-induced intradiscal-pressure measurements should not be directly compared with the leakage failure pressures measured in the present study because of differences in species, specimen preparation, loading method, and endpoint definition.

For comparison with existing annular closure devices, Barricaid provides a clinically relevant reference for bone-anchored annular closure after lumbar discectomy. In a human cadaveric study, Bostelmann et al. (2017) reported an increase in mean intradiscal pressure from 1.01 MPa after discectomy to 1.60 MPa after Barricaid implantation. However, these measurements represent restoration of intradiscal pressure rather than leakage failure pressure and were obtained using a human post-discectomy model with substantially different defect geometry and device fixation. The present study was not designed as a head-to-head comparison with Barricaid; therefore, no conclusions regarding equivalence or superiority can be drawn from numerical values reported across these studies.

The static failure-pressure test used in this study should be interpreted as an assessment of acute annular containment rather than as a comprehensive evaluation of intervertebral disc mechanical function. In the intact disc, compressive loading increases nucleus pulposus pressurization, which is resisted by tensile stresses within the annulus fibrosus. Therefore, the pressure at first visible gel extrusion provides a mechanically relevant index of the ability of an annular repair to resist pressure-driven leakage through the defect. Volumetric injection has been used to generate intradiscal pressurization that is analogous in principle, although not mechanically equivalent, to the pressure increase produced during spinal loading (Lee et al., 2004). In addition, intradiscal pressure has been associated with disc height and axial compliance under controlled loading conditions (Vergroesen et al., 2014). Nevertheless, the pressure measured in the present study was generated by monotonic alginate infusion in an *ex vivo* porcine motion segment rather than by external physiological loading. The higher failure pressures observed in Groups C1 and C2 therefore demonstrate increased acute leakage resistance within the standardized 3-mm defect model, but they do not establish restoration of native disc height, compressive stiffness, range of motion, creep behavior, or long-term mechanical durability. Experimental rupture pressure may be used to characterize susceptibility to pressure-induced disc extrusion (Iencean, 2000); however, static failure pressure cannot predict repair performance under repeated compression, flexion–extension, lateral bending, or axial rotation. Ramped failure testing is primarily relevant to extrusion or herniation resistance, whereas cyclic and range-of-motion testing is required to evaluate functional restoration and fatigue performance (Dixon et al., 2021).

Future studies should evaluate the repair construct under physiologically relevant cyclic and multiaxial loading to determine fatigue durability beyond the acute sealing performance assessed here. A prospective porcine lumbar motion-segment protocol could apply cyclic axial compression between 100 and 700 N at 1 Hz for up to 20,000 cycles, with flexion–extension loading incorporated to challenge the repair under nonaxial conditions. Key outcomes should include leakage onset, graft migration or fixation failure, disc-height loss, compressive stiffness, and residual leakage strength after cyclic loading (Thoreson et al., 2017).

Decellularized soft-tissue matrices have been investigated as potential scaffolds for defect filling because they may retain aspects of the native extracellular-matrix architecture while reducing cellular components (Jiang et al., 2023; Lin et al., 2016). However, clinically relevant challenges, including graft migration, dimensional mismatch, immune response, and inadequate host–graft integration, require further evaluation. In the present study, the processed annulus fibrosus allograft retained its gross structural integrity during rehydration and expanded within the defect, which may have contributed to its resistance to gel leakage. The decellularization procedure used in the present study was based on a previously optimized porcine AF protocol in which substantial DNA reduction and preservation of collagen and glycosaminoglycan content were demonstrated (Wu et al., 2017). However, residual DNA content, histological confirmation of cell removal, and quantitative extracellular-matrix preservation were not independently assessed in the specific graft batches used for the present biomechanical experiments; therefore, batch-specific decellularization efficiency and biological consistency cannot be established from the current data. Nevertheless, this study assessed only immediate biomechanical sealing under static pressurization and did not evaluate cytocompatibility, cellular infiltration, tissue remodeling, biological healing, or long-term integration. Therefore, the present findings support the short-term mechanical feasibility of the repair construct but do not establish its biological or regenerative efficacy. These outcomes should be investigated in future *in vitro* studies and longitudinal *in vivo* studies incorporating histological, immunological, and mechanical assessments.

The 26-gauge puncture model used in this study represents a small, geometrically controlled annular access injury and is most relevant to needle-based intradiscal procedures rather than to the larger and more irregular annular defects that may remain after lumbar discectomy. This model was selected to provide a reproducible proof-of-concept platform for comparing the acute sealing performance of different graft configurations and fixation strategies while minimizing variability in defect size and orientation. Needle-puncture models have previously been used to characterize gauge-dependent annular disruption, leakage pathways, and changes in mechanical integrity (Wang et al., 2007; Yang et al., 2016). In addition, long-term clinical evidence has associated discography exposure with accelerated progression of lumbar disc degeneration (Carragee et al., 2009). Nevertheless, clinical post-discectomy defects may involve annular tissue loss, disruption of multiple lamellae, partial nucleotomy, and irregular defect margins. Accordingly, the present findings should be interpreted as comparative acute containment data for a standardized small annular defect and should not be directly extrapolated to the mechanical performance of the repair in larger post-discectomy defects. Further studies using clinically representative annulotomy or partial-discectomy models under cyclic and multiaxial loading are warranted.

Several limitations of this study should be acknowledged. First, although porcine intervertebral discs share several morphological and biomechanical characteristics with human discs, the failure-pressure values obtained in this *ex vivo* porcine model may not directly translate to human intervertebral discs. In addition, the present study evaluated only acute leakage failure pressure under monotonic pressurization. Therefore, the results do not characterize other aspects of whole-disc mechanical function, including compressive stiffness, range of motion, viscoelastic creep, or fatigue durability under repeated physiological loading.

Second, although specimen source, animal age and weight range, post-mortem interval, storage medium and temperature, injury location, and nominal injury dimensions were standardized, several potentially relevant specimen-specific variables were not independently quantified. Specifically, AF thickness at the injury site, tissue water content, the residual diameter of the 26-gauge puncture after elastic recoil, and the actual achieved injury or defect depth were not measured for each specimen. Because these factors may influence local tissue resistance and intradiscal leakage pressure, they may have contributed to the observed interspecimen variability and reduced the precision of the estimated treatment effects. Variations in local AF thickness and residual defect geometry could alter the effective leakage-path length and the mechanical interference between the graft and surrounding AF, whereas differences in tissue hydration may influence both AF compliance and hydration-induced graft expansion. Likewise, variation in the achieved injury or defect depth could alter communication with the nucleus pulposus and thereby affect the pressure at first visible leakage. Computer-generated random allocation and standardized specimen preparation were used to reduce systematic between-group bias, but these procedures cannot eliminate residual confounding arising from unmeasured biological variation.

Accordingly, the present results should be interpreted as group-level proof-of-concept comparisons rather than precise specimen-normalized estimates of sealing strength. In particular, Group A received a 26-gauge puncture injury and should be regarded as a puncture-reference condition rather than a defect-matched negative control. The most directly comparable evaluations of graft geometry and MPSS fixation are therefore those among Groups B1, B2, C1, and C2, which underwent the same standardized 3-mm defect-preparation protocol. Future studies should include an unrepaired standardized 3-mm AF defect group as a true defect-matched negative control to better isolate the effects of allograft filling and MPSS fixation from those of defect size and geometry. Prospective measurement of AF thickness, hydration-related parameters, and achieved defect dimensions would also allow these variables to be incorporated into the statistical analysis and their contribution to failure pressure to be assessed directly.

Third, this was an *in vitro* study and did not evaluate biological responses such as cellular infiltration, tissue remodeling, immune response, host–graft integration, or long-term healing. A broader *in vivo* animal study is being developed to investigate histological healing, intervertebral disc mechanical properties, and motion behavior over time. Finally, the relatively small sample size of six specimens per group, together with the inherent variability of *ex vivo* biological tissues, limits the precision and statistical power of the present study. Nonsignificant comparisons should therefore not be interpreted as evidence of equivalence between repair methods. Although the direction and magnitude of the principal findings support the proof-of-concept mechanical feasibility of the repair construct, larger, adequately powered studies are required to confirm these effect estimates and improve their generalizability.

## 5 Conclusion

This study demonstrated that, among porcine lumbar motion segments with standardized 3-mm AF defects, MPSS fixation significantly increased acute leakage resistance for both allograft configurations. The hydration-expanded truncated-conical allograft showed a higher mean failure pressure than the standard cylindrical allograft when used without fixation, although this difference did not reach statistical significance after adjustment for multiple comparisons. No significant difference was observed between the two graft geometries when MPSS fixation was applied. These findings support the proof-of-concept mechanical feasibility of the allograft-MPSS construct under static pressurization. Group A should be interpreted only as a puncture-reference condition rather than a defect-matched control. The present findings do not establish equivalence or superiority to commercial annular closure devices or predict long-term performance under physiological cyclic loading.

## Data Availability

The raw data supporting the conclusions of this article will be made available by the authors, without undue reservation.
