# Supplementary material for: A novel allograft combined with modified purse-string suture fixation for annulus fibrosus repair: biomechanical evaluation in a porcine lumbar disc model
Source: Front Bioeng Biotechnol. 2026 Sep 28;14:1738755. doi: 10.3389/fbioe.2026.1738755 (PMC13655539; doi:10.3389/fbioe.2026.1738755)
Supplement: Supplementary file 1 [file Table1.docx]

| Variable | Group A | Groups B1–C2 | Control or data status |
| --- | --- | --- | --- |
| Animal age | 6 months | 6 months | Specimen-selection criterion |
| Animal weight | 80–100 kg | 80–100 kg | Specimen-selection criterion |
| Maximum post-mortem interval | 12 h | 12 h | Standardized |
| Storage medium | 0.9% saline | 0.9% saline | Standardized |
| Storage temperature | 4 °C | 4 °C | Standardized |
| Injury location | Right anterolateral AF | Right anterolateral AF | Standardized |
| Injury instrument | 26-gauge needle | 3-mm drill | Standardized |
| Nominal injury depth | 11–12 mm | 10 mm | Prespecified protocol dimension |
| Nominal defect diameter | Defined by 26-gauge needle | 3 mm | Actual post-creation opening not remeasured |
| Defect-preparation guide | Not applicable | ID 3 mm; OD 5 mm | Standardized guide |
| AF thickness | Not measured | Not measured | Acknowledged limitation |
| Tissue water content | Not measured | Not measured | Saline storage used as operational hydration control |
| Actual achieved depth | Not independently verified | Not independently verified | Acknowledged limitation |
